# Report on the Weather and Diseases of Cincinnati, in the Spring of 1828

**Published:** 1828-06

**Authors:** 


### II. ORIGINAL.

4. *Report on the Weather and Diseases of Cincinnati, in the Spring of* 1828. The mean temperature of Spring, at this place, according to observations made for five years in succession, is 54° 29; which is within the fraction of a degree, of the mean heat of the year. For the period just named, the mean heat of each month was as follows: March 43° 97, April 57° 58, May 61° 32, from which results the mean temperature just stated.

The spring is, generally, supposed to present greater extremes of temperature, than the other seasons; but, at Cincincinnati, they stand, in this respect, in the following order: Winter, Spring, Autumn, Summer. From observations for a a series of years, the thermometer may be expected to fall in March to 16°, and rise to 72o—in April to 28°, and rise to 84°—in May to 40°, and rise to 88°;—and, if it should not descend and ascend to these numbers, it ought to be considered that the variations are unusually moderate.

Our vernal winds are chiefly S. W., N. E., N. W., S. E. and W.—prevailing in the order in which they are here enumerated.

In the number of its fair days, our Spring occupies the following place in the catalogue of the seasons: Summer, Autumn, Spring, Winter.

In its progress, this season appears to be governed by no constant law; for in different years, it presents a very great variety in all its characteristics. Sometimes it is cold and rainy. Our deepest snows are, occasionally, in March; and snow, sometimes, falls, copiously, as late as the middle of May. March is subject to long continued rains, with north east winds; April is, generally, productive of showers, many of which are extremely abundant; May is, almost always, rainy at the close of its first week, and immediately afterwards brings frost. Its latter half is generally hot and dry. Now and then the whole vernal season is dry. It would not be loose phraseology to say, that winter and summer do not much respect the divisions of our calendar; for the former not unfrequently runs far into March, and the latter, still oftener, commences by the 20th of May. On the whole, our Spring is like a sitter, who does not retain the same aspect long enough, to suffer a portrait of definite character to be painted of him.

The Spring of the present year was rather cool and wet, than otherwise. The former quality might have been anticipated, after so mild a winter; but the latter could scarcely have been expected to follow so much rain, as fell in January and February.

Its prevalent diseases were the following: 1. Inflammatory affections of the respiratory apparatus. 2. Measles. 3. Mumps. 4, Ague and Fever. 5. Diarrhœa.

